# Detection of novel CYP11A1-derived secosteroids in the human epidermis and serum and pig adrenal gland

**DOI:** 10.1038/srep14875

**Published:** 2015-10-08

**Authors:** Andrzej T. Slominski, Tae-Kang Kim, Wei Li, Arnold Postlethwaite, Elaine W. Tieu, Edith K. Y. Tang, Robert C. Tuckey

**Affiliations:** 1 Department of Dermatology, University of Alabama at Birmingham, AL, USA; 2 VA Medical Center, Birmingham, AL, USA; 3 Departments of Pharmaceutical Sciences, Memphis, TN, USA; 4 Medicine, University of Tennessee HSC, Memphis, TN, USA; 5 VA Medical Center, Memphis, TN, USA; 6 School of Chemistry and Biochemistry, University of Western Australia, Crawley, WA, Australia

## Abstract

To investigate whether novel pathways of vitamin D3 (D3) and 7-dehydrocholesterol (7DHC) metabolism initiated by CYP11A1 and previously characterized *in vitro*, occur *in vivo*, we analyzed samples of human serum and epidermis, and pig adrenals for the presence of intermediates and products of these pathways. We extracted human epidermis from 13 individuals and sera from 13 individuals and analyzed them by LC/qTOF-MS alongside the corresponding standards. Pig adrenal glands were also analyzed for these steroids and secosteroids. Epidermal, serum and adrenal samples showed the presence of D3 hydroxy-derivatives corresponding to 20(OH)D3, 22(OH)D3, 25(OH)D3, 1,25(OH)_2_D3, 20,22(OH)_2_D3, 20,23(OH)_2_D3, 20,24(OH)_2_D3, 20,25(OH)_2_D3, 20,26(OH)_2_D3, 1,20,23(OH)_3_D3 and 17,20,23(OH)_3_D3, plus 1,20(OH)_2_D3 which was detectable only in the epidermis. Serum concentrations of 20(OH)D3 and 22(OH)D3 were only 30- and 15-fold lower than 25(OH)D3, respectively, and at levels above those required for biological activity as measured *in vitro*. We also detected 1,20,24(OH)_3_D3, 1,20,25(OH)_3_D3 and 1,20,26(OH)_3_D3 in the adrenals. Products of CYP11A1 action on 7DHC, namely 22(OH)7DHC, 20,22(OH)_2_7DHC and 7-dehydropregnenolone were also detected in serum, epidermis and the adrenal. Thus, we have detected novel CYP11A1-derived secosteroids in the skin, serum and adrenal gland and based on their concentrations and biological activity suggest that they act as hormones *in vivo*.

Vitamin D3 (D3) is a product of the photochemical transformation of 7-dehydrocholesterol (7DHC, precursor to cholesterol) after the absorption of ultraviolet B (UVB) energy (280–320 nm) by the unsaturated B ring of the sterol^1^,^2^. In humans, >95% of systemic D3 is produced in the outer layer of the skin, the epidermis, where it serves as a prohormone for conversion to the biologically active 1α,25-dihydroxyvitamin D3 (1,25(OH)_2_D3)^2^,^3^. The D3, formed in the epidermis or provided by the diet, is transported to the liver where it is hydroxylated at C25 by CYP27A1 or CYP2R1 to form 25(OH)D3. The 25(OH)D3 enters the circulation and is then hydroxylated at C1α either in the kidney or peripheral tissues expressing CYP27B1, to form 1,25(OH)_2_D3^4^,^5^,^6^,^7^. This process, D3 → 25(OH)D3 → 1,25(OH)_2_D3, also operates locally in the epidermis^2^,^8^,^9^. 1,25(OH)_2_D3, in addition to regulating calcium metabolism, has important pleiotropic effects that include stimulation of differentiation and inhibition of proliferation of cells of different lineage, anti-cancerogenic effects, stimulation of innate- and inhibition of adaptive- immunity, inhibition of inflammation as well as several other endocrine and developmental effects^4^,^10^,^11^,^12^,^13^,^14^. 1,25(OH)_2_D3 is inactivated by sequential oxidations and shortening of its side chain which starts with hydroxylation at C24, all catalyzed by CYP24A1^5^,^7^,^15^,^16^,^17^.

The traditionally recognised function of CYP11A1 is the catalysis of the sequential hydroxylation of cholesterol (C) followed by cleavage of the side chain to produce pregnenolone (P): C → 22(OH)C → 20,22(OH)_2_C → P^18^,^19^. Recently, evidence has been provided that 7-dehydrocholesterol (7DHC), ergosterol and vitamins D3 and D2 also serve as substrates for CYP11A1^20^,^21^,^22^,^23^,^24^,^25^. 7DHC is a slightly better substrate for CYP11A1 than cholesterol and its side chain cleavage follows a similar sequence producing 7-dehydropregnenolone (7DHP): 7DHC → 22(OH)7DHC → 20,22(OH)_2_7DHC → 7DHP (reviewed in^26^). In contrast, CYP11A1-mediated metabolism of vitamin D involves sequential hydroxylations that start predominantly at C20 or C22, but do not lead to the cleavage of the side chain. Rather, several hydroxy-derivatives of D3 are produced: D3 → 20(OH)D3 + 22(OH)D3 → (OH)_n_D3 (reviewed in^26^,^27^). The major product of this pathway, 20OH)D3, can also serve as a substrate for CYP24A1 and CYP27A1, with CYP24A1 hydroxylating it at C24 or C25 and CYP27A1 hydroxylating it at C25 or C26^28^,^29^,^30^. The products of the above reactions can be further hydroxylated at C1α, by CYP27B1^31^,^32^ with the exception of 17,20,23(OH)_3_D3 which is the final product of CYP11A1 action on D3^25^,^26^.

Products of the novel CYP11A1-initiated secosteroidal pathways exert anti-proliferative, pro-differentiation, and anti-inflammatory effects on cultured skin cells, comparable or better than those of 1,25(OH)_2_D3^33^,^34^,^35^,^36^,^37^,^38^,^39^,^40^,^41^. They show antifibrotic activities, both *in vitro*^37^,^38^,^39^ and *in vivo*, on bleomycin induced scleroderma^39^ and also shows anti-cancer activities that are dependent on the cell-type and lineage^38^,^40^,^41^,^42^,^43^,^44^. The novel hydroxy-derivatives of D3 exert these phenotypic effects acting as biased agonists on the vitamin D receptors (VDR)^27^,^45^ or as reverse agonists on retinoic orphan acid receptors (ROR) α and γ^46^. Thus, we have discovered novel secosteroidal pathways initiated by CYP11A1 and modified by other CYPs for which the intermediates and/or products display biological activity^26^,^27^.

The major product of CYP11A1 action on D3, 20(OH)D3, is noncalcemic at extremely high doses: 30–60 μg/kg in mice^40^,^42^,^47^ and 3 μg/kg (the highest dose tested to date) in rats^44^, without any signs of toxicity. Therefore 20(OH)D3, and perhaps its metabolites, are excellent candidates for primary or adjuvant therapy of hyperproliferative or inflammatory disorders^27^.

The current challenge is to define whether the novel secosteroids are produced *in vivo* from endogenous D3 and can therefore be defined as natural products. Our initial studies using cultured keratinocytes and colon cells, as well as fragments of adrenal glands and placentae, clearly demonstrated that addition of exogenous vitamin D or 7DHC to incubation media results in their transformation to the secosteroidal derivatives in a dose-dependent manner that requires CYP11A1^48^,^49^,^50^,^51^. The above considerations have mandated the testing of whether these compounds are indeed produced *in vivo* from their accumulation in human skin and sera, or in adrenal glands where expression of CYP11A1 is high. In this paper we report for the first time that many of hydroxy-derivatives of vitamin D and products of 7DHC metabolism resulting from the action of CYP11A1, are detectable in the human epidermis and serum and in pig adrenal glands by liquid chromatography-mass spectrometry (LC-MS).

## Results and Discussion

### An overview

To investigate whether the novel pathways of 7DHC (provitamin D3) and D3 metabolism initiated by CYP11A1, defined from *in vitro* studies^26^,^27^, may also be functional *in vivo*, we extracted human epidermis from samples obtained from 13 individuals including 6 African-Americans (AA) and 7 Caucasians (C), and sera from 13 individuals (12C and 1 Hispanic), and analyzed them by LC/qTOF-MS alongside the corresponding standards. These measurements were supplemented by analysis of the pig adrenal gland, an organ expressing high levels of CYP11A1.

### *In vivo* detection of 7DHC metabolites

**Figure 1:**
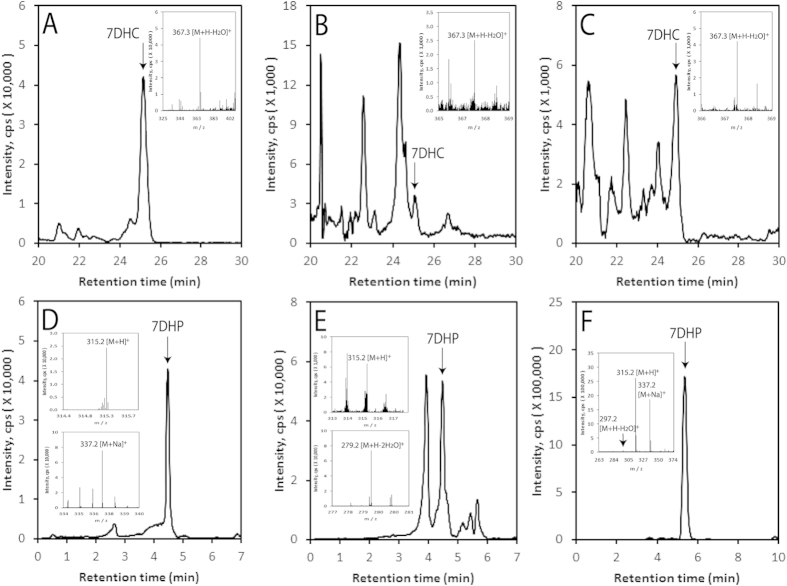
Detection of 7DHC and 7DHP in the human epidermis, human serum and pig adrenals. Extracted ion chromatograms (EIC) are shown for the human epidermis (**A,D**), human serum (**B,E**) and the pig adrenal (**C,F**) using *m/z* = 367.2 [M + H − H_2_O]^+^ for 7DHC (**A–C**) and *m/z* = 337.2 [M + Na]^+^ (**D**), 279.3 [M + H-2H_2_O]^+^ (E) or 315.2 = [M + H]^+^ (**F**) for 7DHP. Arrows indicate the retention times of the corresponding standards. Inserts for panels (**A–C**) show the mass spectra corresponding in retention time of 7DHC while inserts of panels (**D–F**) panels show the mass spectra corresponding to the retention time of 7DHP. Note that the UPLC conditions were different for (**F**) where a longer column was used compared to the other panels (see Materials and Methods).

**Figure 2:**
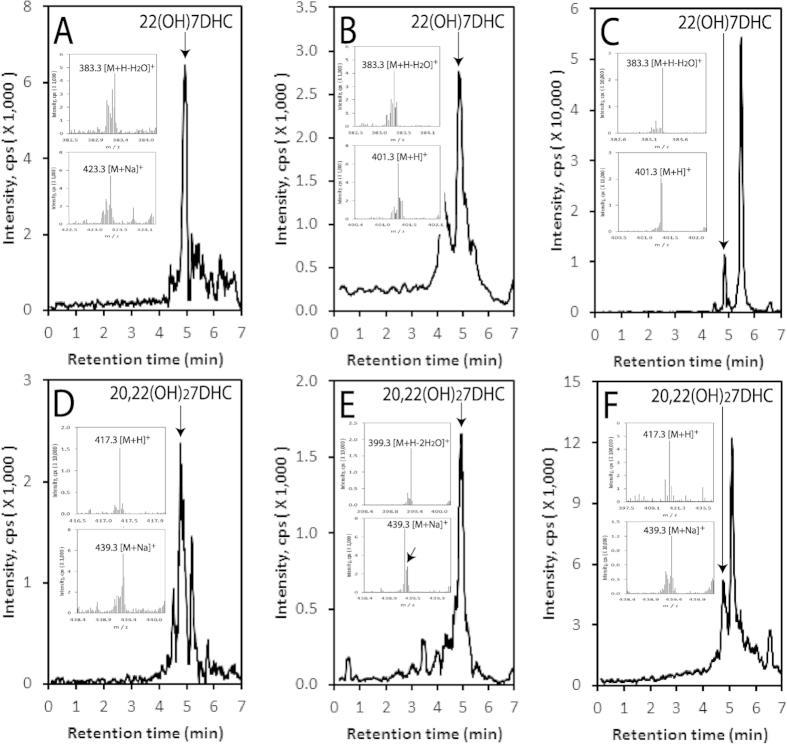
Detection of 22(OH)7DHC and 20,22(OH)_2_7DHC in the pig adrenal, human epidermis and human serum. The LC-MS spectra were measured on fractions with retention times corresponding to either 22(OH)7DHC or 20,22(OH)_2_7DHC that were pre-purified on a Waters C18 column (250 × 4.6 mm, 5 μm particle size) with a gradient of acetonitrile in water (40–100%) (see Materials and Methods). Extracted ion chromatograms are shown for human epidermis (**A,D**), serum (**B,E**) and the pig adrenal (**C,F**), and were measured using *m/z* = 383.3 [M + H − H_2_O]^+^ (**A**) or 401.3 [M + H]^+^ (**B,C**) for 22(OH)7DHC, and m/z = 439.3 [M+Na]^+^ (**D,E**) or *m/z* = 417.3 [M + H]^+^ (**F**) for 20,22(OH)_2_7DHC. Arrows indicate the retention times of the corresponding standards. Inserts show the mass spectra corresponding to the retention time of either 22(OH)7DHC (**A–C**) or 20,22(OH)_2_7DHC (**D–F**).

The analyses of extracted samples using LC/qTOF-MS demonstrate that endogenous 7DHC (Fig. 1A–C) is present in human serum, human epidermis and the pig adrenal gland, as is the product of CYP11A1 action, 7DHP (Fig. 1D–F). Specifically 7DHP-derived species with *m/z* corresponding to 315.2 [M + H]^+^ and 337.2 [M + Na]^+^, and a retention time corresponding to the 7DHP, were detected in the human epidermis. In human serum, 7DHP-derived species displayed *m/z* values of 315.2 [M+H]^+^ and 279.2 [M + H − H_2_O]^+^, while in pig adrenals *m/z* values were 279.2 [M + H − H_2_O]^+^, 315.2 [M+H]^+^ and 337.2 [M + Na]^+^ ] (Fig. 1D–F). The estimated concentration of 7DHP in the epidermis was 36.84 ± 17.14 ng/mg protein and there were no differences in its concentration in relation to sex, age or racial background. These samples were also analyzed for the presence of 22(OH)7DHC and 20,22(OH)_2_7DHC, intermediates in the conversion of 7DHC to 7DHP. Again species with *m/z* and RT corresponding to the 22(OH)7DHC and 20,22(OH)_2_7DHC standards were clearly present in the pig adrenal and human epidermis and serum (Fig. 2). For 22(OH)7DHC *m/z* values of 401.3 [M + H]^+^, 383.3 [M + H − H_2_O]^+^ and/or 423.3 [M + Na]^+^ were observed while for 20,22(OH)_2_7DHC the expected *m/z* values of 417.3 [M + H]^+^, 399.3 [M + H − H_2_O]^+^ and/or 439.3 [M + Na]^+^ were seen. These data substantiate the results of our previous *in vitro* and *ex-vivo* assays 20,21,48,49 and provide important documentation that 7DHC can be metabolized to 7DHP by CYP11A1 *in vivo* , in a sequential manner: (7DHC → 22(OH)7DHC → 20,22(OH)_2_7DHC), with local (epidermis, adrenal gland) and systemic (serum) accumulation of the intermediates and products of the pathway. A future challenge will be to determine whether 7DHP is further metabolized by other steroidogenic enzymes which are expressed in the skin (reviewed in^52^) and whether these ∆7-sterols/steroids undergo UVB-induced transformation to the corresponding secosteroids, as suggested by *in vitro* studies^38^,^53^,^54^,^55^.

### *In vivo* detection of novel vitamin D3 hydroxyderivatives

Following our *in vitro* and *ex-vivo* studies on the transformation of exogenous vitamin D3 to hydroxy-derivatives (with 20(OH)D3 being the major metabolite) by cells and tissues expressing CYP11A1^50^, we tested the human and pig samples for the presence of the novel mono-, di- and tri-hydroxy-derivatives of D3 in comparison to 25(OH)D3 and 1,25(OH)_2_D3. It should be noted that for the precise detection of vitamin D hydroxy-derivatives, they were first separated on Waters C18 column (250 × 4.6 mm, 5 μm particle size) with a gradient of acetonitrile in water (40–100%). Fractions with RTs corresponding to specific standards were then analyzed by UPLC on an Agilent Zorbax Eclipse Plus C18 column (2.1 × 50 mm, 1.8 μm particle size) with a gradient of methanol in water (see Materials and Methods), connected to a Xevo™ G2-S qTOF.

**Figure 3:**
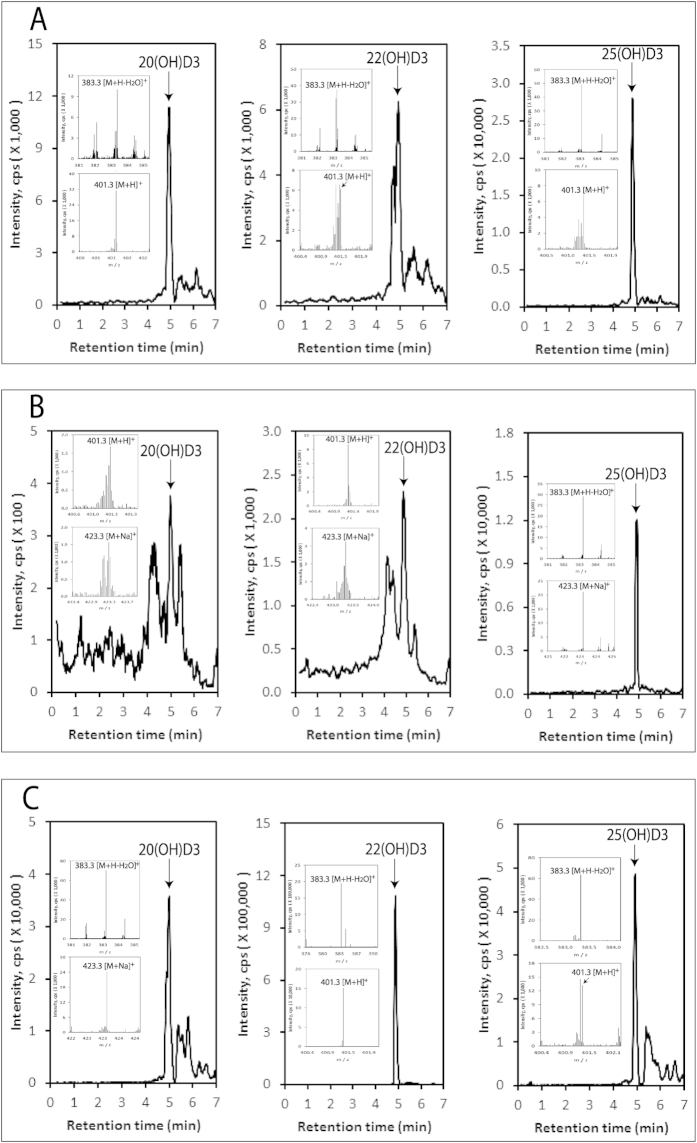
Detection of 20(OH)D3, 22(OH)D3 and 25(OH)D3 in the human epidermis, human serum and the pig adrenal gland. The LC-MS spectra were measured on fractions that were pre-purified on a Waters C18 column (250 × 4.6 mm, 5 μm particle size) with a gradient of acetonitrile in water (40–100%) as in Fig. 2. EICs are shown for epidermis (**A**), serum (**B**) and the pig adrenal gland (**C**) and were measured using *m/z* = 383.3 [M + H − H_2_O]^+^ for (**A,C**) and 25(OH)D3 in (**B**) or 401.3 [M + H]^+^ for 20(OH)D3 and 22(OH)D3 in (**B**). The identified peaks had RTs corresponding to either the 20(OH)D3, 22(OH)D3 or 25(OH)D3 standards, indicated by arrows. The inserts show the mass spectra of samples corresponding to the retention times of the standards.

Monohydroxy-D3 species were detected in the epidermis, serum and adrenal glands with the extracted ion chromatogram (EIC) analyzed using *m/z* = 383.3 [M + H − H_2_O]^+^, with retention times corresponding to the 20(OH)D3, 22(OH)D3 and 25(OH)D3 standards (Fig. 3). Additional ions detected at RTs of the corresponding standards included 401.3 [M + H]^+^ and/or 423.3 [M + Na]^+^ for the epidermis, serum and adrenals, (Fig. 3, inserts), further supporting the identification of 20(OH)D3, 22(OH)D3 and 25(OH)D3 in these samples. This detection of 20(OH)D3 and 22(OH)D3 *in vivo* is in agreement with our previous demonstration that cells or tissues expressing CYP11A1 are able to hydroxylate exogenous vitamin D3 in positions C20 or C22^50^. Quantification of the concentrations of these monohydroxy-D3 metabolites in the epidermis and serum showed that there is a significantly higher concentration of 25(OH)D3 in human serum than 20(OH)D3 and 22(OH)D3 (30 and 15 folds, respectively). The concentration of 25(OH)D3 in the epidermis was lower than that of 20(OH)D3 (Table 1). These differences indicate that the classical pathway of D3 activation (D3 → 25(OH)D3) is the major one with respect to the systemic level of vitamin D metabolites, because of the massive production of 25(OH)D3 in the liver. However, the higher concentration of 20(OH)D3 than 25(OH)D3 in the epidermis may arise from it being produced at a greater rate, or alternatively, it being metabolised more slowly than 25(OH)D3. Both possibilities require further experimental testing using skin organ culture. Preliminary analysis of levels of these metabolites for gender, age and racial group showed no statistical difference in epidermal concentration of either 20(OH)D3 or 25(OH)D3 (Supplemental Fig. 1). Similarly there were no differences for these parameters in human serum except for significantly higher levels of 25(OH)D3 in older individuals than younger ones (Supplemental Fig. 2). The higher levels of 25(OH)D3 in the serum of older subjects than younger ones may result from higher compliance by senior individuals to include an oral supplementation of D3.

**Figure 4:**
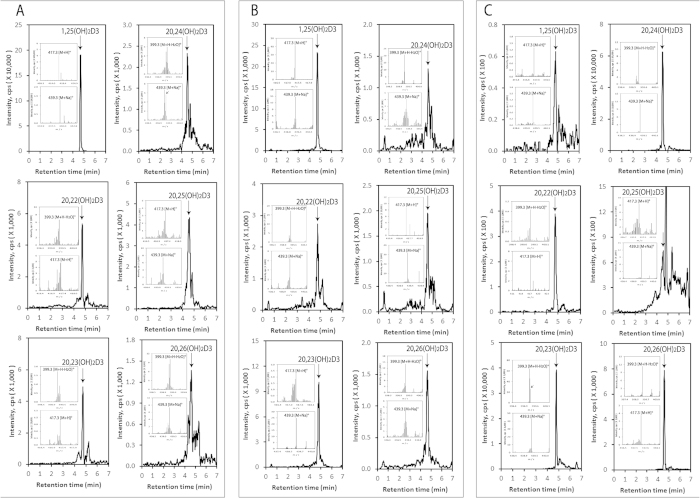
Novel dihydroxyvitamin D3 metabolites can be detected in the human epidermis, human serum and the pig adrenal gland. The LC-MS spectra were measured on fractions that were pre-purified on Waters C18 column (250 × 4.6 mm, 5 μm particle size) as in Fig. 2. EICs are shown for epidermis (**A**), serum (**B**) and the pig adrenal (**C**) and were measured using *m/z* = 399.3 [M + H − H_2_O]^+^ for 20,22(OH)_2_D3 and 20,23(OH)_2_D3 in A, and 1,25(OH)_2_D3, 20,22OH)_2_D3, 20,24(HO)_2_D3 and 20,26(OH)_2_D3 in C; 417.3 [M + H]^+^ for 1,25(OH)_2_D3 in (**A**) and 20,23(OH)_2_D3 and 20,25(HO)_2_D3 in C; 439.3 [M + Na]^+^ for 20,24(OH)_2_D3, 20,25(OH)_2_D3 and 20,26(OH)_2_D3 in (**A,B**). Arrows indicate the retention times of the corresponding standards. The mass spectra of samples corresponding to the retention time of each dihydroxyvitamin D3 standard are shown in the inserts.

The human and pig samples were further analyzed for the presence of dihydroxyvitamin D3 metabolites. Dihydroxy-D3 species were detected in the epidermis, serum and adrenal glands with the EIC analyzed using *m/z* = 399.3 [M + H − H_2_O]^+^ (Fig. 4). Identified metabolites with retention times corresponding to authentic standards were 1,25(OH)_2_D3, 1,20(OH)_2_D3, 20,22(OH)_2_D3, 20,23(OH)_2_D3, 20,24(OH)_2_D3, 20,25(OH)_2_D3 and 20,26(OH)_2_D3. 1,20(OH)_2_D3 was also detected in the epidermis (data not shown) but was below the level of detection in the serum and adrenal. The mass spectra of samples taken at the retention times of the authentic standards (inserts, Fig. 4) showed the expected ions at 417.3 [M + H]^+^ and 439.3 [M + Na]^+^ as well as the 399.3 ion, further confirming the identity of products as species of dihydroxyvitamin D3. It should be noted that the pre-purification of the secosteroids in the sample by HPLC prior to LC-MS (see Fig. 4 legend) was required to separate 20,22(OH)_2_D3 from 20,23(OH)_2_D3, 20,24(OH)_2_D3 from 20,25(OH)_2_D3, and 20,26(OH)_2_D3 and 1,20(OH)_2_D3 from the background. We also analyzed adrenal extracts directly by UPLC-MS without this pre-purification step and detected dihydroxy-D3 ions at 417.3 [M + H]^+^, 399.3 [M + H − H_2_O]^+^ and 439.3 [M + Na]^+^ with RTs corresponding to 1,25(OH)_2_D3 or 20,22(OH)_2_D3, 20,23(OH)_2_D3 or 20,24(OH)_2_D3, 20,25(OH)_2_D3 and 20,26(OH)_2_D3 (not shown), which further confirms the endogenous accumulation of these metabolites in the adrenal gland.

**Figure 5:**
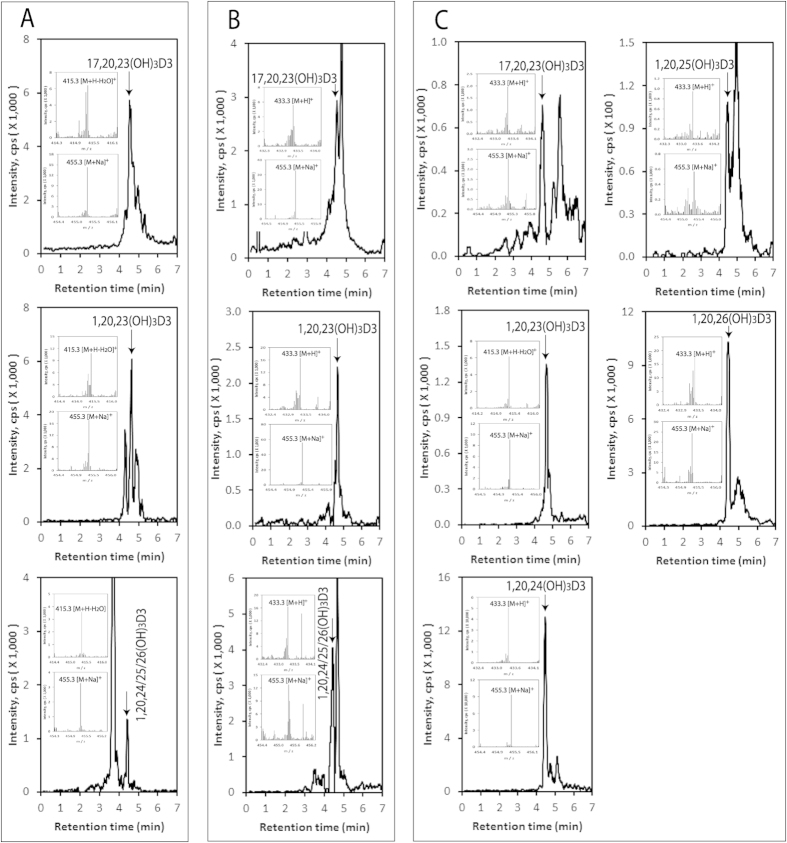
Novel trihydroxy-vitamin D3 metabolites are also present in in the human epidermis, human serum and pig adrenal. The LC-MS spectra were measured on fractions which were pre-purified on a Waters C18 column (250 × 4.6 mm, 5 μm particle size), as in Fig. 2. EICs are shown for the epidermis (**A**), serum (**B**), adrenal (**C**) and were measured using *m/z* = 415.3 [M + H − H_2_O]^+^ except 17,20,23(OH)3D3 in (**C**) (455.3 [M + Na]^+^). Each standard is identified by arrow and the mass spectra of samples corresponding to the retention time of each trihydroxyvitamin D3 standard are shown in the inserts.

Novel trihydroxy-D3 metabolites were detected in the epidermis, serum and pig adrenal glands with the EICs being measured using *m/z* = 433.3 [M + H]^+^ (Fig. 5). Identified trihydroxyvitamin D3 species with retention times corresponding to authentic standards included 17,20,23(OH)_3_D3 and 1,20,23(OH)_3_D3. Besides the 433.3 ion, the mass spectra (Fig. 5 inserts) also gave ions with *m/z* values of 415.3 [M + H − H_2_O]^+^ and/or 455.3 [M + Na]^+^, further supporting their identification as trihydroxyvitamin D3 species. We also detected a peak in the epidermal and serum extracts corresponding to the single RT displayed by 1,20,24(OH)_3_D3, 1,20,25(OH)_3_D3 and 1,20,26(OH)_3_D3 (unseparated by LC under the conditions used (see Materials and Methods)) (Fig. 5A,B, lower panels). Pre- separation of these secosteroids by HPLC on a Waters C18 column (250 × 4.6 mm, 5 μm particle size) with a gradient of acetonitrile in water (40–100%) prior to LC-MS provided insufficient sample to detect the individual trihydroxyvitamin D3 species in serum and the epidermis. However, we could detect all three compounds in the adrenal extracts by this procedure (Fig. 5C).

The above results show that in addition to classical 25(OH)D3 and 1,25(OH)_2_D3, CYP11A1-derived monohydroxxy-, dihydroxy- and trihydroxy-D3 metabolites are present in the human epidermis and serum, and in pig adrenals. Detection and quantification of 20(OH)D3 in human serum and the epidermis using LC/qTOF-MS has substantiated our initial finding of the presence of 20(OH)D3 in the serum^50^ and epidermis^56^ using LC-MS/MS. Detection of 22(OH)D3 in these samples represents a new finding demonstrating that D3 can be hydroxylated either at C20 or C22 *in vivo* for local or systemic use, by tissues or organs expressing CYP11A1, consistent with *in vitro* and *ex-vivo* data^21^,^26^,^50^,^57^. While 22(OH)D3 shows significantly lower biological activity than 20(OH)D3 towards skin cells^57^, its higher concentration in human serum mandates future testing of its phenotypic potency towards other cell types to determine whether it displays a cell-type dependent role in physiology or pathology. Detection of 20,22(OH)_2_D3, 20,23(OH)_2_D3, 20,24(OH)_2_D3, 20,25(OH)_2_D3, 20,26(OH)_2_D3, 1,20,23(OH)_3_D3 and 17,20,23(OH)_3_D3 in the epidermis and human serum and pig adrenal gland is of great significance, because it shows that *in vivo* D3 can be hydroxylated in a sequential fashion by CYP11A1 following the sequence: D3 → 20(OH)D3 + 22(OH)D3 → 20,22(OH)_2_D3 + 20,23(OH)_2_D3 → 17,20,23(OH)_3_D3 as predicted theoretically^26^ and consistent with *in vitro* data^21^,^25^,^50^,^57^. Furthermore the hydroxy-derivatives identified illustrate that 20(OH)D3 can be further metabolized by CYP27A1 and CYP24A1, or liver microsomes, as documented *in vitro*^28^,^29^,^58^. The latter indicates that the following pathway of 20(OH)D3 metabolism occurs *in vivo* that is independent of CYP11A1: 20(OH)D3 → 20,24(OH)_2_D3 + 20,25(OH)_2_D3 + 20,26(OH)_2_D3. The detection of 1,20(OH)_2_D3 and 1,20,23(OH)_3_D3 in some of the samples analyzed demonstrates that CYP11A1-derived D3 hydroxymetabolites are further hydroxylated by CYP27B1, the only enzyme with appreciable 1α–hydroxylase activity, substantiating our previous *in vitro*^31^,^32^ and *ex vivo* data^50^. The absence of 1,20(OH)_2_D3 in the human serum suggests that *in vivo,* 1α-hydroxylation of 20(OH)D3 occurs predominantly at the local level, which is consistent with low catalytic efficiency of CYP27B1 toward 20(OH)D3 or 20(OH)D2^32^,^40^,^59^. We also detected species corresponding to 1,20,24(OH)_3_D3, 1,20,25(OH)_3_D3 and 1,20,26(OH)_3_D3 in the adrenal extracts. These results suggest that products of CYP24A1 or CYP27A1 hydroxylation of 20(OH)D3 may be further hydroxylated at C1α, at least in the adrenal gland, which does expresses CYP27B1^50^. This pathway is consistent with the high catalytic efficiency that purified CYP27B1 displays towards 20,24(OH)_2_D3, 20,25(OH)_2_D3 and 20,26(OH)_2_D3^32^.

The 1α-hydroxy-derivatives of 20(OH)D3 and 20(OH)D2^40^,^60^ and of 20,23(OH)_2_D3 inhibit keratinocyte proliferation^31^ with similar potency to that of 1,25(OH)_2_D3^27^. Detection of 17,20,23(OH)_3_D3 in the epidermis, serum and adrenal glands deserves special attention since it represents the final product of CYP11A1 dependent metabolism of D3 and it is not a substrate for CYP27B1^32^ and is probably poorly metabolized by CYP27A1 and CYP24A1^26^. Of note, preliminary studies demonstrate that 17,20,23(OH)_3_D3 is biologically active in skin cells^27^,^39^,^45^ suggesting that it may have a role in biological regulation, perhaps exerted in a tissue- and cell lineage- dependent fashion.

### Concluding remarks and perspectives

In this study we show for the first time the presence of CYP11A1-derived products of 7DHC metabolism (22(OH)7DHC, 20,22(OH)_2_7DHC and 7DHP) and D3 metabolism (20(OH)D3, 22(OH)D3, 20,22(OH)_2_D3, 20,23(OH)_2_D3 and 17,20,23(OH)_3_D3) in the human epidermis and serum, and the pig adrenal gland. Our data also indicate that some of the CYP11A1-derived secosteroids can be acted on by CYP27B1 (producing 1,20(OH)_2_D3 and 1,20,23(OH)_3_D3) or CYP27A1 and/or CYP24A1 (producing 20,24(OH)_2_D3, 20,25(OH)_2_D3 and 20,26(OH)_2_D3). These findings complement and are consistent with previous *in vitro* and *ex-vivo* studies on the CYP11A1-initiated metabolism of D3^20^,^23^,^25^,^27^,^28^,^29^,^31^,^32^,^50^,^57^,^58^ and 7DHC^20^,^23^,^48^,^49^ by demonstrating that these pathways do operate *in vivo*, and they are further modified by other CYPs metabolizing D3 or steroids, as predicted from *in vitro* analyses^26^. In the skin, CYP11A1-derived ∆7-sterols/steroids are likely to be transformed into the corresponding secosteroids after exposure to UVB^21^,^37^,^38^, the significance of which represents a future challenge to explore. Importantly, the major metabolites of these novel steroid/secosteroidogenic pathways display anti-proliferative, prodifferentiation and anti-inflammatory activities in a cell-lineage dependent fashion^27^,^33^,^34^,^35^,^37^,^38^,^39^,^41^,^43^,^44^,^48^,^56^,^57^. Thus these pathways, as well as their intermediates and/or products, are likely play a role in the regulation of physiological process and their related pathology. This is supported by the relatively high concentrations of 22(OH)D3 and 20(OH)D3 in the plasma, levels only 15–30 fold lower than 25(OH)D3 and therefore higher than 1,25(OH)_2_D3, and at the concentration levels required to see effects *in vitro*. This further suggests that the measurement of the major CYP11A- derived hydroxy-derivatives of D3 in the human serum may be necessary to fully assess vitamin deficiency or sufficiency, as opposed to a single measurement of 25(OH)D3. Some of these new secosteroids are non-toxic and non-calcemic at relatively high doses, as demonstrated for 20(OH)D3^42^,^44^,^47^ and 20,23(OH)_2_D3^39^, suggesting that they are not only endogenous bioregulators, but also potential therapeutics (primary or adjuvant) for the treatment of inflammatory and/or hyperproliferative skin disorders resistant to 1,25(OH)_2_D3 action. In conclusion, the detection of novel CYP11A1-derived secosteroids in the skin, adrenal gland and serum in conjunction with their biological activity suggest that they act as hormones *in vivo* for which the effects would depend on their local and systemic production, and their metabolism.

## Materials and Methods

### Source of steroids and secosteroids

7DHC, vitamin D3, 25(OH)D3, and 1,25(OH)_2_D3 were obtained from Sigma-Aldrich (St. Louis, MO). 7DHP was synthesized as described previously^38^,^53^, while 22(OH)7DHC and 20,22(OH)_2_7DHC 20(OH)D3 were produced from 7DHC enzymatically using purified bovine CYP11A1^49^. 20(OH)D3, 22(OH)D3, 20,22(OH)_2_D3, 20,23(OH)_2_D3 and 17,20,23(OH)_3_D3 were produced from vitamin D3 using purified CYP11A1, while 1,20(OH)2D3 was similarly produced from 1α(OH)D3^23^,^25^,^57^,^60^. 20,24(OH)_2_D3, 20,25(OH)_2_D3 and 20,26(OH)_3_D3, were produced from 20(OH)D3 using recombinant CYP27A1 or CYP24A1^28^,^29^. 1,20,24(OH)_3_D3, 1,20,25(OH)_3_D3 and 1,20,26(OH)_3_D3 were made by by 1α-hydroxylation of either 20,24(OH)_2_D3, 20,25(OH)_2_D3 or 20,26(OH)_2_D3 using recombinant CYP27B1^32^. These compounds were purified by reverse-phase HPLC, and their structures and purities were determined by NMR and mass spectrometry^23^,^25^,^28^,^29^,^32^,^36^,^48^,^49^,^57^,^60^.

### Use of tissues and serum samples

The experiments were performed in accordance with relevant guidelines (see below) and the experiments were approved by the Institutional Review Board (IRB) (Human Subject Assurance Number 00002301) and the Institutional Animal Care and Use Committee (IACUC)(Animal Welfare Assurance Number A3325–01) at the University of Tennessee Health Science Center (UTHSC).

The use of human skin and cells was approved by the IRB at the UTHSC as an exempt protocol #4 (Dr. A. Slominski, P.I.). The protocol was classified for exempt status under 45CFR46.102 (f) in that is does not involve “human subjects” as defined therein. Informed consent is waived in accord with 45CFR46.116(d). The research involved no more than minimal risk, it will not adversely affect the right and welfare of the subjects, and it could practicably be carried out without the waiver. Material in this category consists of tissues that are left over, or in excess of what is needed, for pathological diagnosis or analysis. Condition (4) at 45CFR46.116(d) is not applicable to this study. Human skin samples (n = 13) from both males (n = 7) and females (n = 6), 30 to 90 years old of African-American (AA; n = 6) and Caucasian (C, n = 7) races were obtained from the Regional One Health Center and the Methodist University Hospital, Memphis, TN.

The collection of human serum was approved by the IRB protocol #7526 (Dr. A. Postlethwaite, P.I.). An informed consent was obtained from all subjects involved in this study. Human serum was collected from 13 volunteers (12 Caucasians and 1 Hispanic), 25–61 years old including 10 females and 3 males following protocols described previously^50^.

Collection of pig adrenals was approved by the UTHSC Institutional Animal Care and Use Committee (IACUC), which has the Animal Welfare Assurance Number A3325–01. Pig adrenals were collected from a female Landrace cross Large White pig, 2 years old following protocols approved by IACUC as described previously^50^.

### Extraction of vitamin D3, 7DHC and their metabolites from tissues and serum

Extraction of D3, 7DHC and their metabolites from tissues and serum followed protocols described previously^49^,^50^ with some modifications^61^. Briefly, the epidermis was separated from dermis and processed as described in^61^. The epidermal tissue was homogenized in PBS followed by a second homogenization in 75% acetonitrile. After centrifugation the supernatants were filtered and dried. The human serum samples were extracted with methanol:water (9:1) with vortexing. The precipitated proteins were removed by centrifugation, the supernatants filtered using a syringe filter (PES, 0.45 μm, 30 mm; Celltreat, Shirley, MA) and then dried using a speedvac drier (Savant instruments, Inc. Holbrook, NY). Pieces of pig adrenal glands were suspended in PBS and homogenized following the addition of 2.5 volumes of methylene chloride. Samples were centrifuged and the supernatants dried as above. The dried extracts were stored at −80 °C until further analyses.

### Detection of vitamin 7DHC and vitamin D3 metabolites

The above extracts were analyzed by liquid chromatography and mass spectrometry (LC-MS) as described previously^49^,^50^,^51^,^61^ with some modifications. In most cases (as indicated in the Figure legends), to minimize the interference from other molecules present in the samples, we initially pre-purified samples by HPLC using a long column (Waters C18 column, 250 × 4.6 mm, 5 μm particle size). Elution was carried out with a gradient of acetonitrile in water (40–100%) at a flow rate of 0.5 ml/min (15 min), followed by a wash with 100% acetonitrile for 30 min at a flow rate of 0.5 ml/min and for 20 min at a flow rate of 1.5 ml/min. Fractions with retention times (RTs) corresponding to the secosteroids of interest, which were well separated under these conditions (RTs decreasing from mono-, di- to trihydroxylated), were collected separately. These fractions were then subjected to UPLC (Waters ACQUITY I-Class UPLC (ultra-performance liquid chromatography) system (Waters, Milford, USA)) on an Agilent Zorbax Eclipse Plus C18 column (2.1 × 50 mm, 1.8 μm particle size), which was connected to a Xevo™ G2-S qTOF (quadrupole hybrid with orthogonal acceleration time-of-flight) tandem mass spectrometer (Waters, Milford, USA)^51^,^61^. For the UPLC, a gradient of methanol in water containing 0.1% formic acid (20–60% for 3 min, 60–100% for 1 min, 100% for 2.1 min, 100–20% for 0.1 min), at flow rate of 0.3 ml/min, was used. Using this short column and under the UPLC conditions employed, these metabolites showed similar but distinct retention times (as indicated in Fig. 2). For 7DHP detection in the skin and serum, the initial HPLC step was omitted and the samples were directly analyzed by LC-MS using the UPLC conditions described above. Similarly, 7DHC and vitamin D3 were directly analyzed by LC-MS but a Waters Atlantis dC18 column (100 × 4.6 mm, 5 μm particle size) was used with a gradient of methanol in water (85–100%) containing 0.1% formic acid for 20 min followed by 100% methanol containing 0.1% formic acid for 10 min, at a flow rate of 0.5 ml/min. These conditions were also used for measuring 7DHP in the pig adrenal (results shown in Fig. 1F).

For MS analysis the scan range was 50 to1000 Da in the positive mode, and all data were collected in the centroid mode. The capillary and cone voltages were 3.0 kV and 30 V, respectively. The desolvation gas was maintained at 1000 L/h at a temperature of 500 °C. The cone gas was 100 L/h with a source temperature of 150 °C. The data acquisition rate was 0.3 s, with a 20 second interval. The lockspray frequency was every 20 s using Leucine Enkephalin solution (100 ng/mL) as the lockspray reference compound (m/z 556.2771) with a flow rate of 5 μL/min. The MS data were collected in the full scan mode with low (6 V) and high (ramp from 20 V to 40 V) collision energy (CE) data channels to get both the parent ions (MS) and the daughter ions (MS/MS). All data were acquired and processed by Waters MassLynx v4.1 software.

For quantification of 20(OH)D3, 22(OH)D3 and 25(OH)D3, LC/qTOF-MS was used. The UPLC of extracts was carried out with a Waters Atlantis dC18 column (100 × 4.6 mm, 5 μm particle size) with a gradient of methanol in water (85–100%) containing 0.1% formic acid for 20 min followed by 100% methanol containing 0.1% formic acid for 10 min, at a flow rate of 0.5 ml/min. An Agilent Zorbax Eclipse Plus C18 column (2.1 × 50 mm, 1.8 μm) was used to quantify 7DHP and 17,20,23(OH)_3_D3 in extracts using a gradient of methanol in water containing 0.1% formic acid (20–60% for 3 min, 60–100% for 1 min, 100% for 2.1 min, 100–20% for 0.1 min), at flow rate of 0.3 ml/min. MS analysis was performed as described above. The concentrations of 7DHP and secosteroids in the epidermis and serum were calculated from MS peak areas in relation to standards curves generated using the corresponding standards at m/z = 315.2 [M + H]^+^ for 7DHP, 383.3 [M + H − H_2_O]^+^ for 20(OH)D3, 22(OH)D3 and 25(OH)D3, and 455.3 [M + Na]^+^ for 17,20,23(OH)_3_D3. The values are presented as means ± SE in ng per mg protein or ml serum.

### Statistical analysis

Data are presented as means ± SE and were analyzed using the Student’s t-test, using Microsoft Excel and Prism 4.00 (GraphPad Software, San Diego, CA). Statistically significant differences are denoted in tables and figures.

## Additional Information

**How to cite this article**: Slominski, A. T. *et al.* Detection of novel CYP11A1-derived secosteroids in the human epidermis and serum and pig adrenal gland. *Sci. Rep.* **5**, 14875; doi: 10.1038/srep14875 (2015).

## Supplementary Material

Supplementary Information

## Figures and Tables

**Table 1:** Serum and tissue content of mono-hydroxy D3 metabolites.

Secosteroid	Epidermis (ng/mgprotein)	Serum (ng/ml)
20(OH)D3	0.40 ± 0.15	1.15 ± 0.20
22(OH)D3	ND*	2.38 ± 0.65
25(OH)D3	0.06 ± 0.02	33.58 ± 5.36
D3	0.13 ± 0.05	2.39 ± 0.34

^*^ND, not done because the 22(OH)D3 signal is buried in the contaminating peaks.
