# Supplementary material for: Detection of novel CYP11A1-derived secosteroids in the human epidermis and serum and pig adrenal gland
Source: Sci Rep. 2015 Oct 8;5:14875. doi: 10.1038/srep14875 (PMC4597207; doi:10.1038/srep14875)

Supplementary Information

Detection of novel CYP11A1-derived secosteroids in the human epidermis and serum and pig adrenal gland

Andrzej T. Slominski[1,2], Tae-Kang Kim[1], Wei Li[3], Arnold Postlethwaite[4.5], Elaine W. Tieu[6], Edith K. Y. Tang[6] and Robert C. Tuckey[6]

[1]Department of Dermatology, University of Alabama at Birmingham, [2]VA Medical Center, Birmingham, AL, USA

Departments of [3]Pharmaceutical Sciences and [4]Medicine, University of Tennessee HSC, [5]VA Medical Center, Memphis, TN, USA;

[6]School of Chemistry and Biochemistry, UWA, Crawley, WA, Australia

Supplemental figure 1. Concentrations of 20(OH)D3 and 25(OH)D3 in the epidermis in relation to race, gender and age of the donor. For quantification of 20(OH)D3 and 25(OH)D3 LC/qTOF-MS was used with sample analysis performed on a Waters Atlantis dC18 column (100 × 4.6 mm, 5 µm particle size) as described in the Materials and Methods. The upper right panels show representative LC-MS chromatograms of 20(OH)D3 and 25(OH)D3 in the epidermis The concentrations of the products were calculated from MS peak areas in relation to standard curves generated using the corresponding standards at m/z = 383.3 $[M+H-H_2O]^+$. The individual values and the means are presented. Data were analyzed using student's t-test with *p*<0.05 considered as significant. The 22(OH)D3 content was not analyzed because its signal was buried in a contaminating peak.

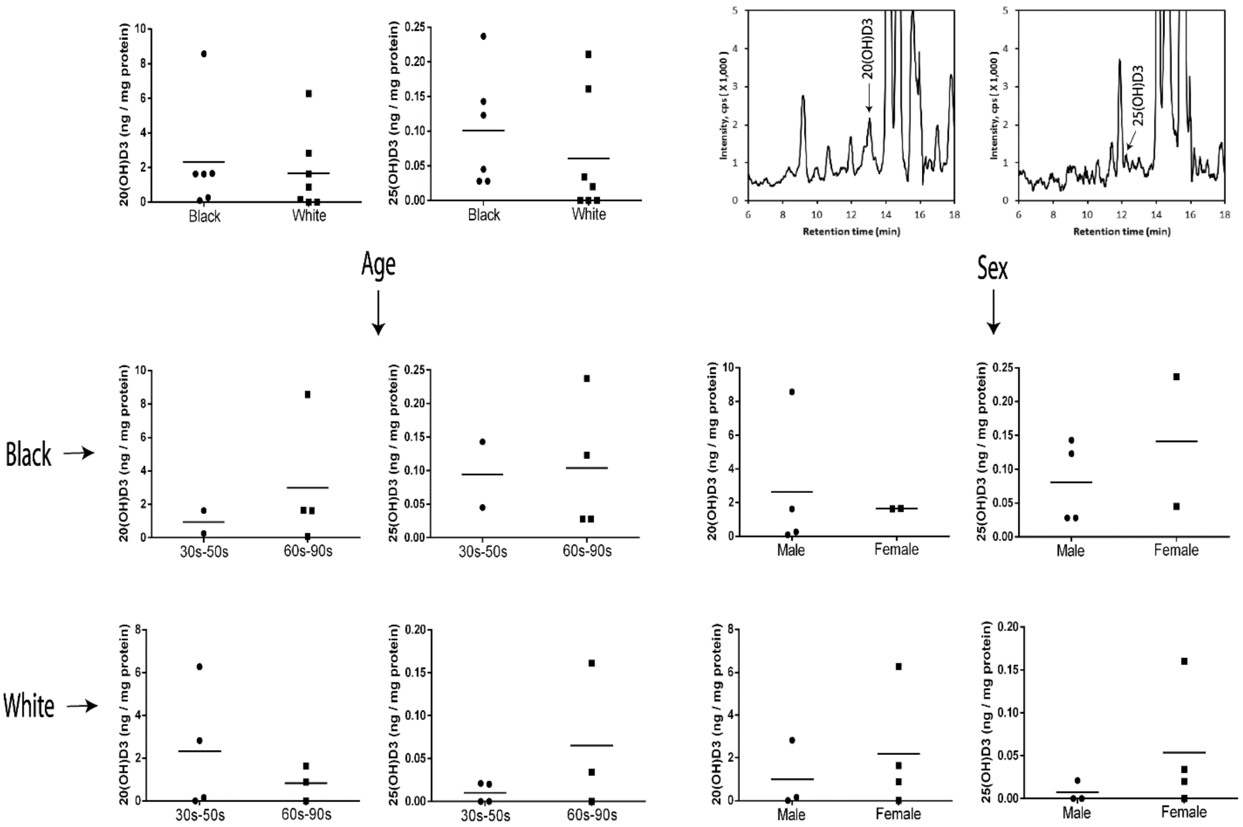

Supplemental figure 2. Concentrations of 20(OH)D3, 22(OH)D3 and 25(OH)D3 in serum in relation to gender and age of the donor. The quantification of 20(OH)D3, 22(OH)D3 and 25(OH)D3 was performed) as described in supplemental figure 1 and the Materials and Methods. The upper panels show representative LC-MS chromatograms of 20(OH)D3, 22(OH)D3 and 25(OH)D3 in the human serum. The individual values and the means are presented. Data were analyzed using student's t-test, * *p*<0.05

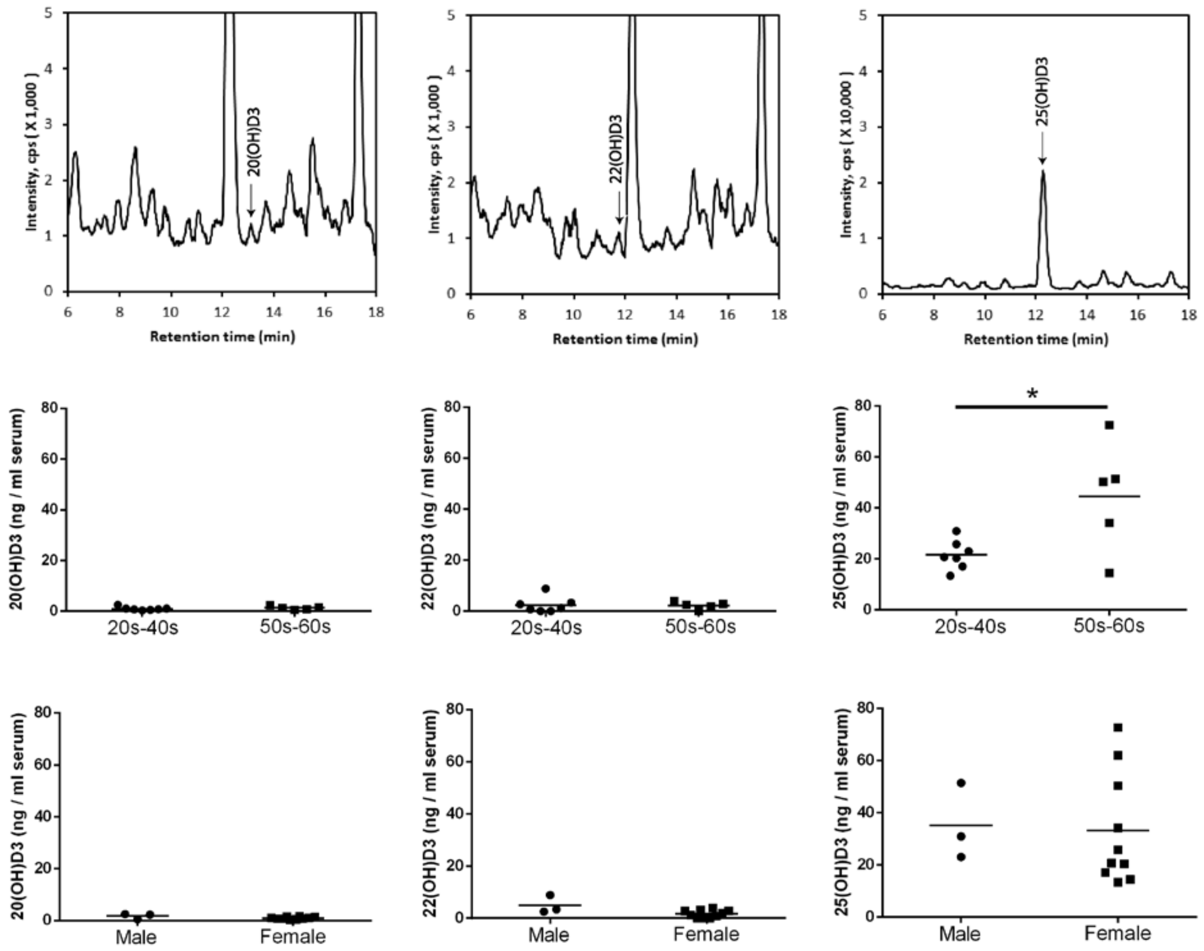

Supplement: Supplementary Information [file srep14875-s1.pdf]
